# Differences in blood flow dynamics between balloon- and self-expandable valves in patients with aortic stenosis undergoing transcatheter aortic valve replacement

**DOI:** 10.1186/s12968-023-00970-9

**Published:** 2023-10-26

**Authors:** Yuki Takahashi, Kiwamu Kamiya, Toshiyuki Nagai, Satonori Tsuneta, Noriko Oyama-Manabe, Takeshi Hamaya, Sho Kazui, Yutaro Yasui, Kohei Saiin, Seiichiro Naito, Yoshifumi Mizuguchi, Sakae Takenaka, Atsushi Tada, Suguru Ishizaka, Yuta Kobayashi, Kazunori Omote, Takuma Sato, Yasushige Shingu, Kohsuke Kudo, Satoru Wakasa, Toshihisa Anzai

**Affiliations:** 1 Department of Cardiovascular Medicine, Faculty of Medicine and Graduate School of Medicine, Hokkaido University, Kita 15, Nishi 7, Kita-Ku, Sapporo, Hokkaido 060-8638 Japan; 2 Department of Diagnostic and Interventional Radiology, Hokkaido University Hospital, Kita 14, Nishi 5, Kita-Ku, Sapporo, Hokkaido 060-8648 Japan; 3 Department of Radiology, Jichi Medical University Saitama Medical Center, 1-847 Amanuma-Cho, Omiya-Ku, Saitama-City, Saitama 330-8503 Japan; 4 Department of Cardiovascular Surgery, Faculty of Medicine and Graduate School of Medicine, Hokkaido University, Kita 15, Nishi 7, Kita-Ku, Sapporo, Hokkaido 060-8638 Japan

**Keywords:** Four-dimensional flow magnetic resonance imaging, Aortic stenosis, Transcatheter aortic valve replacement, Blood flow dynamics

## Abstract

**Background:**

The differences in pre- and early post-procedural blood flow dynamics between the two major types of bioprosthetic valves, the balloon-expandable valve (BEV) and self-expandable valve (SEV), in patients with aortic stenosis (AS) undergoing transcatheter aortic valve replacement (TAVR), have not been investigated. We aimed to investigate the differences in blood flow dynamics between the BEV and SEV using four-dimensional flow cardiovascular magnetic resonance (4D flow CMR).

**Methods:**

We prospectively examined 98 consecutive patients with severe AS who underwent TAVR between May 2018 and November 2021 (58 BEV and 40 SEV) after excluding those without CMR because of a contraindication, inadequate imaging from the analyses, or patients’ refusal. CMR was performed in all participants before (median interval, 22 [interquartile range (IQR) 4–39] days) and after (median interval, 6 [IQR 3–6] days) TAVR. We compared the changes in blood flow patterns, wall shear stress (WSS), and energy loss (EL) in the ascending aorta (AAo) between the BEV and SEV using 4D flow CMR.

**Results:**

The absolute reductions in helical flow and flow eccentricity were significantly higher in the SEV group compared in the BEV group after TAVR (BEV: − 0.22 ± 0.86 vs. SEV: − 0.85 ± 0.80, *P* < 0.001 and BEV: − 0.11 ± 0.79 vs. SEV: − 0.50 ± 0.88, *P* = 0.037, respectively); there were no significant differences in vortical flow between the groups. The absolute reduction of average WSS was significantly higher in the SEV group compared to the BEV group after TAVR (BEV: − 0.6 [− 2.1 to 0.5] Pa vs. SEV: − 1.8 [− 3.5 to − 0.8] Pa, *P* = 0.006). The systolic EL in the AAo significantly decreased after TAVR in both the groups, while the absolute reduction was comparable between the groups.

**Conclusions:**

Helical flow, flow eccentricity, and average WSS in the AAo were significantly decreased after SEV implantation compared to BEV implantation, providing functional insights for valve selection in patients with AS undergoing TAVR. Our findings offer valuable insights into blood flow dynamics, aiding in the selection of valves for patients with AS undergoing TAVR. Further larger-scale studies are warranted to confirm the prognostic significance of hemodynamic changes in these patients.

## Introduction

Transcatheter aortic valve replacement (TAVR) is performed worldwide for treatment of patients with symptomatic severe aortic stenosis (AS). TAVR improves the clinical outcomes in patients with severe AS, and the indication for this procedure is expanding to younger, lower surgical risk patients [1–3]. In fact, 18% of patients who underwent TAVR had low surgical risk in Japan [4].

Two major types of transcatheter heart valves (THVs) are currently available, the balloon-expandable Edwards SAPIEN3^®^ (Edwards Lifesciences, Irvine, CA, USA) and self-expandable Medtronic CoreValve^®^/Evolut^®^ (Medtronic, Minneapolis, Minnesota, USA) valves. These THVs have structural differences in the stent and valve attachment site. In particular, the self-expandable valve (SEV) is a supra-annular valve designed by placing the attachment site of the prosthetic valve above the tissue annulus, while the balloon-expandable valve (BEV) is an intra-annular valve. The SEV has been shown to ensure a larger effective orifice area (EOA) compared to the BEV, thereby reducing prosthesis–patient mismatch after TAVR [5]. BEV is reportedly associated with lower rates of stroke, paravalvular leakage (PVL), and new pacemaker implantation, whereas the SEV has a lower residual mean transaortic pressure gradient (MPG) [2, 3]. However, the SOLVE-TAVI study showed the noninferiority of the BEV and SEV in terms of the primary efficacy composite endpoint of all-cause death, stroke, PVL, and new pacemaker implantation [6]. Therefore, from a long-term perspective, there are few useful indicators for selecting the appropriate type of THV for patients undergoing TAVR.

Time-resolved three-dimensional (3D) phase-contrast cardiovascular magnetic resonance (CMR), known as the four-dimensional (4D) flow CMR, is a blood flow dynamics imaging modality that allows accurate visualization and quantification of the vascular blood flow dynamics [7]. 4D flow CMR can also be used to quantitatively evaluate wall shear stress (WSS), which is the friction force at the vessel wall due to blood flow, and energy loss (EL), which is the amount of energy dissipated by turbulent kinetic energy and viscous friction in blood flow [8, 9]. Several studies have shown various blood flow patterns, assessed using the 4D flow CMR, in patients with AS undergoing surgical aortic valve replacement or TAVR [10–13]. We previously reported that TAVR improved blood flow dynamics in the ascending aorta (AAo), and a significant negative correlation was observed between the systolic EL in the AAo and EOA index (EOAI) after TAVR [13]. However, the differences in pre- and early post-procedural blood flow dynamics between the types of THVs have not been investigated. Therefore, this study aimed to investigate differences in pre- and early post-procedural blood flow dynamics between BEV and SEV in patients with severe AS undergoing TAVR using 4D flow CMR and to clarify their functional significance.

## Methods

### Study design

This single-center, observational, prospective study included consecutive patients with symptomatic severe AS who underwent TAVR according to current guidelines [14]. The study protocol was approved by the Ethics Committee of Hokkaido University Hospital (018-0223 and 019-0090). The investigation conformed to the principles outlined in the Declaration of Helsinki. All patients provided written informed consent to participate in the study.

### Study population

**Fig. 1:**
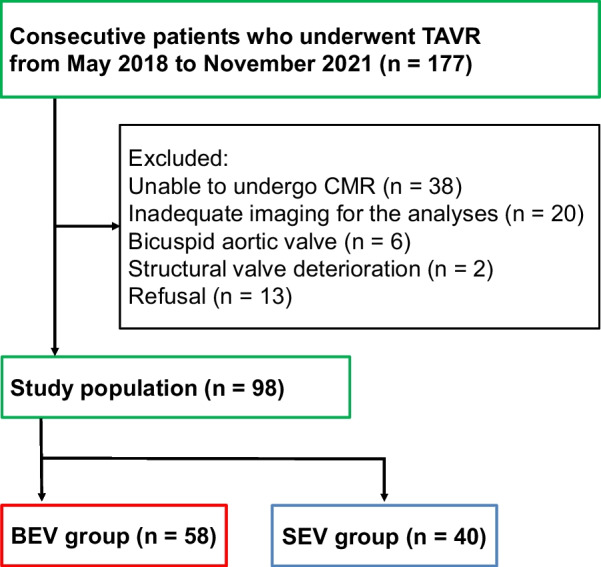
Flow diagram of the present study. *BEV* balloon-expandable valve, *CMR* cardiac magnetic resonance, *SEV* self-expandable valve, *TAVR* transcatheter aortic valve replacement

We initially screened 177 consecutive patients with severe AS who underwent TAVR between May 2018 and November 2021 and met any of the following criteria on transthoracic echocardiography: peak aortic velocity (Vmax) ≥ 4.0 m/s, MPG ≥ 40 mmHg, aortic valve area (AVA) ≤ 1.0 cm^2^, or aortic valve area index (AVAI) ≤ 0.6 cm^2^/m^2^. Of these, the patients who did not undergo CMR due to a contraindication (n = 38) and those with imaging unsuitable for the analysis because of poor image quality due to motion and/or respiration artifact (n = 20) were excluded. Six patients with bicuspid aortic valve and two with AS due to structural valve deterioration after surgical aortic valve replacement were excluded because these patients have abnormal flow patterns compared to those with native tricuspid aortic valve [11, 15, 16]. Thirteen patients refused to participate in this study. Ultimately, 98 patients (58 with BEVs and 40 with SEVs) were included in this study (Fig. 1).

### TAVR procedure

All cases were discussed for the indication of TAVR and selection of the THV type at a meeting of the multidisciplinary Heart Team, as recommended by the current guidelines [1]. We selected the appropriate THV type based on anatomical considerations (e.g., AVA), and electrocardiographic features (e.g., presence of bundle branch block). During the study period, we did not consider 4D flow CMR features for the selection of THV type. TAVR was performed through a transfemoral, transapical, or transsubclavian approach depending on the pre-procedural vascular assessment. In most patients, TAVR was performed with transesophageal echocardiographic guidance under general anesthesia. Pre- and post-dilatations were performed at the operator’s discretion.

### Echocardiography measurements

Echocardiography was performed within 2 weeks before and after TAVR. The following left ventricular (LV) systolic and diastolic parameters were assessed using echocardiography before and after TAVR: LV end-diastolic dimension (LVDD), LV end-systolic dimension, LV ejection fraction (LVEF), ratio between early and late diastolic transmitral flow velocity (E/A), ratio of maximal early diastolic filling wave velocity to maximal early diastolic myocardial velocity (E/e’), and left atrial volume index (LAVI). The LVEF was measured from the apical 4- and 2-chamber images using the biplane method of disks. The left atrial (LA) volume was measured from the standard apical 4-chamber views at end-systole immediately before the mitral valve opening. The biplane method of disks was used to calculate LA volume. Left ventricular mass was calculated according to the following formula:

$${\text{LV mass}}\, = \,0.{8}\, \times \,\left\{ {{1}.0{4}\, \times \,\left[ {\left( {{\text{LVDD}}\, + \,{\text{LV posterior wall thickness}}\, + \,{\text{interventricular septum thickness}}} \right)^{{3}} {-}\left( {{\text{LVDD}}} \right)^{{3}} } \right]} \right\}\, + \,0.{6} \,{\text{g}}{.}$$

LAVI and LV mass index (LVMI) were calculated by dividing the LA volume and LV mass by the body surface area of patients, respectively. The relative wall thickness was defined as two times the posterior wall thickness divided by the LVDD. Left ventricular remodeling was assessed based on the LVDD, LVMI, and relative wall thickness.

### Contrast enhanced computed tomography

All patients underwent ECG-gated contrast enhanced computed tomography (CT) using a 320-row area detector CT (Aquilion ONE ViSION Edition, Toshiba Medical Systems, Otawara, Japan) prior to TAVR to measure aortic annulus area, perimeter, diameter of the AAo, and aortic angle as well as to determine the appropriate THV size and access site of TAVR. All measurements were performed using 3mensio Structural Heart (version 7.0; Structural Heart, Pie Medical Imaging, Maastricht, The Netherlands).

### CMR and data analysis

CMR imaging and data analysis were performed, as described elsewhere [13]. CMR imaging using a 3.0-T scanner (Achieva TX, Philips Healthcare, Best, The Netherlands) with a 32-channel phased-array receiver torso-cardiac coil was performed in patients before (median interval, 22 [interquartile range (IQR) 4–39] days) and after (median interval, 6 [IQR 3–6] days) TAVR. 4D flow CMR data were acquired as sagittal oblique 3D data including the entire heart and thoracic aorta without a contrast agent. The scan parameters were as follows: echo time = 1.73 ms, repetition time = 3.2 ms, flip angle α = 10°, field of view 400 × 400 mm, matrix 256 × 229, in-plane spatial resolution = 1.6 × 1.8 mm^2^, slice thickness = 4 or 5 mm, temporal resolution = 12 phases/cardiac cycle, k-space segmentation factor = 6, and sensitivity encoding factor R = 3. The velocity encoding timing was TR-interleaved. Partial k-space coverage methods and k-t undersampling were not used. Velocity encoding (VENC) was set to individually appropriate values based on the peak blood flow velocity in the AAo with a secured margin. In this study, considering the extremely high peak velocity in the aortic valve of patients with AS, the VENC was set at that value plus 100 cm/s or more, and the actual VENC was confirmed to be 160–450 cm/s (median 300 [IQR 250–310] cm/s) before TAVR and 120–500 cm/s (median 250 [IQR 220–300] cm/s) after TAVR. VENC correction was not performed because of the secured margin in the VENC setting. Acquisition time of the 4D flow CMR ranged from approximately 8–20 min.

In all patients, the LV and aortic hemodynamics were evaluated using commercially available software (iTFlow, Cardio Flow Design Inc., Japan), which visualized the cardiovascular geometry and blood flow [17, 18]. WSS and EL were calculated using this software [18]. Moreover, the blood flow pattern in the AAo was evaluated, as described in a previous study [10]. Peak flow velocity was set below 1 m/s in the whole AAo because blood flow jets in patients with AS are seen only immediately around the aortic valve, while blood flow velocity decreases distal to the aortic valve as the pressure gradient decreases [19]. Two readers (one cardiologist and one radiologist) simultaneously observed blood flow from the patient's left front. Discordant cases were evaluated by a third reader (a radiologist experienced in cardiovascular imaging). Vortical flow was defined as revolving particles around a point within the vessel with a rotation direction deviating by more than 90° from the physiological flow direction [10]. Helical flow was defined as regional fluid circulation along the longitudinal axis of the vessel, thereby creating a corkscrew-like motion [10]. The systolic blood flow pattern was semi-quantitatively evaluated as three grades for the vortical and helical flows: 1 = none (none or almost none), 2 = moderate (obvious. between 1 and 3), and 3 = marked (mainstream). Three analysis planes were positioned perpendicular to the aortic wall at the level of the sinotubular junction (slice 1), mid AAo (slice 2), and proximal to the brachiocephalic trunk (Slice 3) [13]. The peak velocity blood flow eccentricity in the mid AAo (slice 2) during systole was semi-quantitatively evaluated as three grades: 1 = none (if the high-velocity systolic flow was centrally focused, occupying the majority of the vessel lumen), 2 = mild (if the high-velocity systolic flow occupied between one- and two-thirds of the vessel lumen), and 3 = marked (if the high-velocity systolic flow occupied one-third or less of the vessel lumen) [13].

WSS in the AAo was calculated, as described previously [13, 18]. In brief, an anatomical segmentation of the AAo was obtained using the iTFlow software. Subsequently, we determined 3D WSS over the complete AAo and displayed with a color-coded map. The peak WSS was defined as the highest WSS value in this color-coded map of the entire AAo in all cardiac cycles. As the average WSS, the mean value of WSS of the entire AAo in the phase in which the peak was recorded is used. Furthermore, to measure changes in the WSS in each region, measurements of WSS were performed for 12 segments along the aortic circumference for each analysis plane (slice 1 to slice 3) [13].

EL was calculated from the spatial velocity gradient of the blood flow and blood viscosity according to the following formula [9, 18].

$${\text{EL}} = \int {({\upmu })\sum\nolimits_{ij} {\frac{1}{2}\left( {\frac{{\partial u_{i} }}{{\partial \chi_{j} }} + \frac{{\partial u_{j} }}{{\partial x_{i} }}} \right)dV} } ,$$

µ: viscosity of the blood (μ = 0.004 Pa·s). x: horizontal direction of phase image. u: horizontal direction component of blood velocity vector.

The EL across the region of interest (left ventricle or AAo) was calculated for each of the 12 phases/cardiac cycles and averaged for systolic and diastolic phases. The left ventricle was defined as the region from the mitral valve to the aortic valve, and the AAo was defined as the region from the aortic valve to the brachiocephalic artery.

### Statistical analysis

Continuous variables are presented as mean ± standard deviation when normally distributed and as median and interquartile range when not normally distributed. Comparisons between the BEV and SEV groups were performed using the Mann–Whitney U-test for continuous variables and the chi-squared test for categorical variables. Changes in the blood flow dynamic parameters after TAVR were evaluated using the Wilcoxon signed-rank test. An analysis of covariance (ANCOVA) was performed to increase the robustness of the results. We compared changes in blood flow patterns, WSS, and EL between the BEV and SEV groups using an ANCOVA model with the individual pre-TAVR values as covariates. Inter-reader agreement for blood flow patterns was assessed using quadratic weighted kappa statistics (along with their standard errors). All tests were two tailed, and a *P* value < 0.05 was considered statistically significant. All analyses were performed using Stata/IC (version 16; Stata Corp, College Station, TX, USA).

## Results

### Patient characteristics

Baseline characteristics of all the participants are shown in Table 1. There were no significant differences in terms of age, sex, Society of Thoracic Surgeons predicted risk of mortality (STS-PROM) scores, and past histories between the BEV and SEV groups. Parameters related to cardiac function were comparable between the groups, with similar LV dimensions and ejection fractions. The echocardiographic data after TAVR are shown in Table 2. The stroke volume index (SVI) and MPG were higher in the BEV group than those in the SEV group, while there were no significant differences in the other echocardiographic parameters including the degree of PVL and EOAI between the groups.

**Table 1:** Baseline characteristics

Variable	BEV (n = 58)	SEV (n = 40)	*P* value
Age, years	83.7 ± 4.6	84.4 ± 5.1	0.34
Male, n (%)	21 (36)	14 (35)	0.90
Body mass index, kg/m^2^	22.7 ± 4.2	21.5 ± 3.5	0.18
STS-PROM score, %	4.9 (3.6–7.3)	5.3 (4.2–8.5)	0.24
Past history, *n* (%)			
Hypertension	47 (81)	33 (83)	0.85
Dyslipidemia	35 (60)	17 (43)	0.082
Diabetes mellitus	19 (33)	9 (23)	0.27
Atrial fibrillation	11 (19)	7 (18)	0.85
Coronary artery disease	22 (38)	16 (40)	0.84
Stroke	9 (16)	7 (18)	0.79
Laboratory data			
Hemoglobin, g/dL	11.4 ± 1.5	11.7 ± 1.4	0.29
Creatinine, mg/dL	0.81 (0.70–1.15)	0.89 (0.71–1.11)	0.64
NT-proBNP, pg/mL	1089 (483–1787)	1392 (620–3826)	0.064
Echocardiography			
LVDD, mm	45.5 (42.0–49.0)	44.5 (41.0–49.0)	0.86
LVEF, %	68 (56–72)	67 (57–70)	0.41
SVI, mL/m^2^	47.7 ± 12.2	49.0 ± 10.8	0.19
IVS, mm	11.9 ± 2.1	11.9 ± 2.3	0.60
LVMI, g/m^2^	115.4 (100.4–144.4)	120.2 (98.9–141.5)	0.74
LAVI, mL/m^2^	50.0 (39.8–62.5)	49.4 (42.9–69.0)	0.48
E/A ratio	0.63 (0.55–0.76)	0.67 (0.58–1.07)	0.038
E/e’ ratio	14.4 ± 5.1	17.6 ± 7.1	0.042
AVA, cm^2^	0.67 ± 0.15	0.67 ± 0.18	0.83
AVAI, cm^2^/m^2^	0.46 ± 0.12	0.48 ± 0.21	0.98
MPG, mmHg	49 (40–60)	50 (39–71)	0.53
Computed tomography			
Aortic annular area, mm^2^	411 (383–474)	422 (366–458)	0.28
Aortic annular perimeter, mm	72.9 (70.3–78.7)	74.2 (69.3–76.9)	0.37
Ascending aortic diameter, mm	32.7 ± 3.2	32.9 ± 3.2	0.94
Aortic angle, degree	50.8 ± 9.2	47.1 ± 6.2	0.038
Access route for TAVR, *n* (%)			
Femoral	51 (88)	31 (77.5)	< 0.001
Apical	5 (9)	–	
Subclavian	0 (0)	9 (22.5)	
Direct aortic	2 (3)	0 (0)	
THV model, n (%)			
SAPIEN 3^®^	58 (100)	–	
Evolut R^®^	–	14 (35)	
Evolut PRO^®^	–	10 (25)	
Evolut PRO plus^®^	–	16 (40)	
THV size, *n* (%)			
20 mm	2 (3)	–	< 0.001
23 mm	33 (57)	1 (2.5)	
26 mm	19 (33)	18 (45)	
29 mm	4 (7)	19 (47.5)	
34 mm	–	2 (5)	

Continuous variables are presented as mean ± standard deviation if normally distributed, and median (interquartile range) if not normally distributed. Categorical variables are presented as number of patients (%)

*AVAI* aortic valve area index, *BEV* balloon-expandable valve, *IVS* interventricular septum, *LAVI* left atrial volume index, *LVDD* left ventricular end-diastolic dimension, *LVEF* left ventricular ejection fraction, *LVMI* left ventricular mass index, *mPG* mean transaortic pressure gradient, *NT-proBNP* N-terminal pro-brain natriuretic peptide, *SEV* self-expandable valve, *STS-PROM* Society of Thoracic Surgeons Predicted Risk of Mortality, *SVI* stroke volume index, *TAVR* transcatheter aortic valve replacement, *THV* transcatheter heart valve

**Table 2:** Echocardiographic data after TAVR

Variable	BEV (n = 58)	SEV (n = 40)	*P* value
LVDD, mm	46.0 (42.0–49.0)	46.0 (41.0–50.0)	0.89
LVEF, %	67 (62–71)	66 (57–71)	0.35
SVI, mL/m^2^	49.6 ± 12.3	41.9 ± 9.5	0.002
IVS, mm	11.8 ± 2.2	11.7 ± 2.1	0.69
LVMI, g/m^2^	111.0 (97.7–143.6)	122.7 (101.0–139.6)	0.50
LAVI, mL/m^2^	51.1 (36.2–64.3)	55.2 (44.0–64.7)	0.37
E/A ratio	0.71 (0.65–0.87)	0.77 (0.58–1.02)	0.82
E/e’ ratio	16.0 ± 5.2	17.0 ± 6.5	0.62
EOA, cm^2^	1.54 ± 0.36	1.58 ± 0.29	0.50
EOAI, cm^2^/m^2^	1.05 ± 0.26	1.09 ± 0.24	0.38
MPG, mmHg	13 (11–17)	8 (6–11)	< 0.001
PVL, *n* (%)			
None	8 (14)	3 (7.5)	0.12
Mild	42 (72)	24 (60)	
Moderate	8 (14)	13 (32.5)	
Severe	0 (0)	0 (0)	

Continuous variables are presented as mean ± standard deviation if normally distributed, and median (interquartile range) if not normally distributed. Categorical variables are presented as number of patients (%)

PVL paravalvular leakage, other abbreviations are as in Table 1

### Blood flow pattern and flow eccentricity in the ascending aorta

**Fig. 2:**
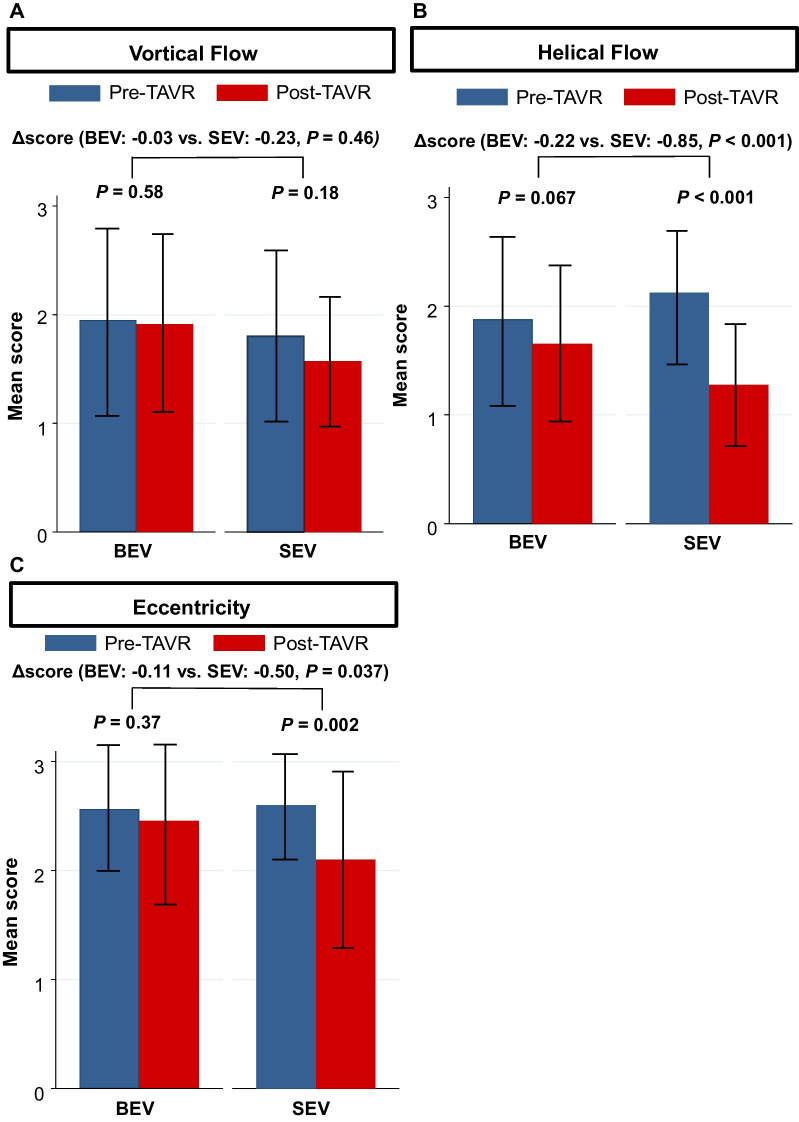
Mean scores of the (**A**) vortical flow, (**B**) helical flow, and (**C**) blood eccentricity before and after TAVR in the BEV and SEV. *BEV* balloon-expandable valve, *SEV* self-expandable valve, *TAVR* transcatheter aortic valve replacement

**Fig. 3:**
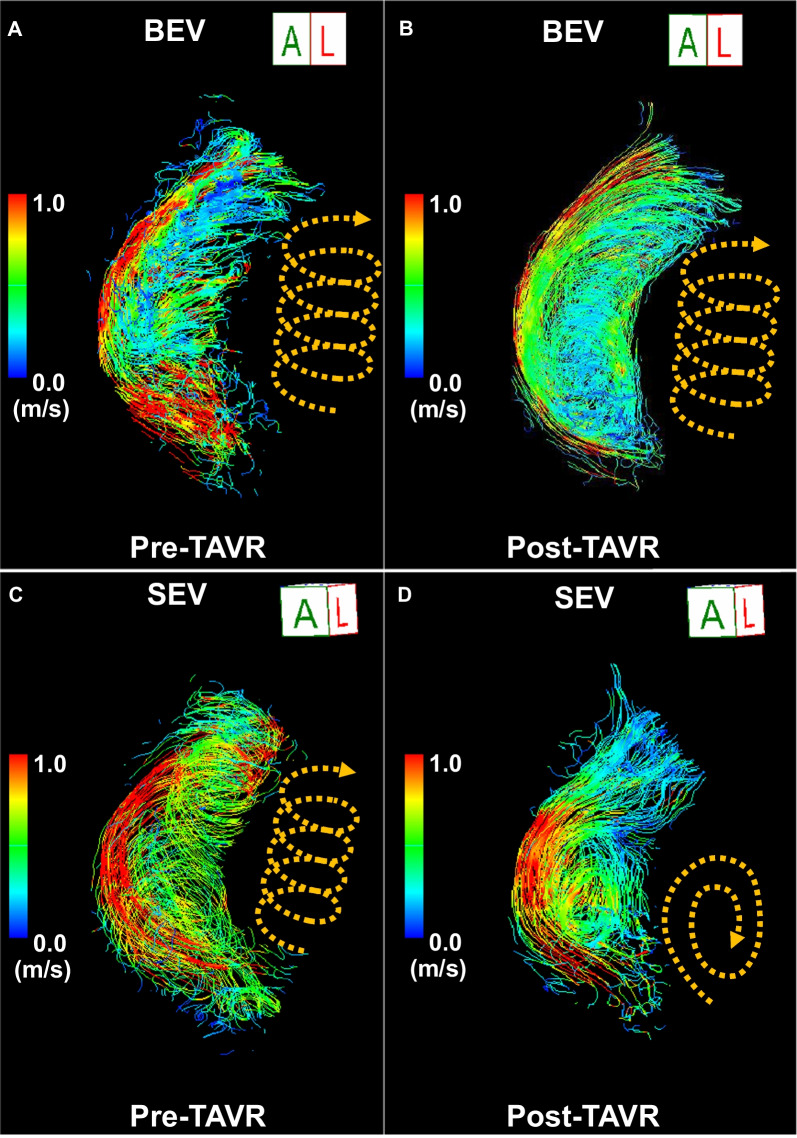
Representative cases. In a patient with an implanted BEV, (**A**) marked helical flow was observed before TAVR, and (**B**) the helical flow did not change significantly after TAVR. In a patient with an implanted SEV, (**C**) marked helical flow was observed before TAVR, and (**D**) the helical flow disappeared after TAVR. BEV, balloon-expandable valve; SEV, self-expandable valve; TAVR, transcatheter aortic valve replacement

The 4D flow CMR parameters before and after TAVR are shown in Tables 3 and 4, respectively. There were no significant differences in flow pattern and flow eccentricity before TAVR between the groups. Changes in blood flow pattern and flow eccentricity in the AAo before and after TAVR are shown in Fig. 2. The mean scores of helical flow and flow eccentricity in the SEV group significantly decreased after TAVR (helical flow score: from 2.12 ± 0.61 to 1.28 ± 0.51, *P* < 0.001, flow eccentricity score: from 2.60 ± 0.50 to 2.10 ± 0.78, *P* = 0.002). Changes in the mean scores of helical flow (BEV: − 0.22 ± 0.86 vs. SEV: − 0.85 ± 0.80, *P* < 0.001) and flow eccentricity (BEV: − 0.11 ± 0.79 vs. SEV: − 0.50 ± 0.88, *P* = 0.037) after TAVR were significantly higher in the SEV group compared to those in the BEV group. There were no significant differences in the degrees of the vortical flow before and after TAVR. In the ANCOVA analysis, the changes in mean scores of the vortical flow, the helical flow, and flow eccentricity were significantly higher in the SEV group compared to those in the BEV group (Table 5). Representative cases of the changes in the blood flow pattern before and after TAVR for the BEV and SEV are shown in Fig. 3. The quadratic weighted kappa coefficients for inter-observer agreement for blood flow patterns were as follows: k = 0.67; standard error = 0.07 for vortex, k = 0.63; standard error = 0.07 for helix, and k = 0.69; standard error = 0.08 for eccentricity.

**Table 3:** 4D flow measurements before TAVR

Variable	BEV (n = 58)	SEV (n = 40)	*P* value
Vortical flow score	1.95 ± 0.85	1.80 ± 0.79	0.44
Helical flow score	1.88 ± 0.77	2.12 ± 0.61	0.071
Flow eccentricity score	2.56 ± 0.60	2.60 ± 0.50	0.99
Average WSS, Pa	6.8 (5.9–8.4)	6.8 (5.7–8.4)	0.62
Peak WSS, Pa	53.5 (48.1–62.2)	53.4 (42.1–65.3)	0.73
Systolic EL (AAo), mW	23.9 (17.2–34.4)	18.9 (9.6–32.9)	0.084
Diastolic EL (AAo), mW	6.9 (3.9–10.5)	3.6 (1.3–9.6)	0.014
Systolic EL (LV), mW	3.8 (2.7– 6.4)	3.1 (1.4–6.3)	0.062
Diastolic EL (LV), mW	5.1 (3.2–9.0)	4.0 (2.0–6.5)	0.045

Continuous variables are presented as mean ± standard deviation if normally distributed, and median (interquartile range) if not normally distributed

4D four-dimensional, AAo ascending aorta, CMR cardiovascular magnetic resonance, EL energy loss, LV left ventricle, WSS wall shear stress, other abbreviations are as in Table 1

**Table 4:** 4D flow measurements after TAVR

Variable	BEV (n = 58)	SEV (n = 40)	*P* value
Vortical flow score	1.91 ± 0.80	1.58 ± 0.64	0.040
Helical flow score	1.66 ± 0.74	1.28 ± 0.51	0.009
Flow eccentricity score	2.45 ± 0.73	2.10 ± 0.78	0.022
Average WSS, Pa	6.0 (5.3–7.0)	4.8 (3.9–5.9)	< 0.001
Peak WSS, Pa	51.2 (40.6–58.7)	41.6 (30.4–53.6)	0.021
Systolic EL (AAo), mW	14.0 (10.7–22.9)	11.6 (4.5–22.7)	0.087
Diastolic EL (AAo), mW	5.7 (3.6–11.8)	4.3 (1.3–12.6)	0.15
Systolic EL (LV), mW	5.1 (2.8–8.1)	3.1 (1.2–7.6)	0.085
Diastolic EL (LV), mW	6.6 (3.3–9.7)	3.3 (1.3–8.0)	0.005

Continuous variables are presented as mean ± standard deviation if normally distributed, and median (interquartile range) if not normally distributed

Abbreviations are as in Tables 1 and 3

**Table 5:** Changes of 4D flow CMR parameters before and after TAVR

Parameter	BEV (n = 58)	SEV (n = 40)	*P* ^a^ value	Mean difference ^b^	*P* ^c^ value
Vortical flow score	− 0.03 ± 1.09	− 0.23 ± 0.97	0.46	− 0.32 (− 0.63 to − 0.02)	0.036
Helical flow score	− 0.22 ± 0.86	− 0.85 ± 0.80	< 0.001	− 0.44 (− 0.70 to − 0.17)	0.0014
Flow eccentricity score	− 0.11 ± 0.79	− 0.50 ± 0.88	0.037	− 0.37 (− 0.67 to − 0.07)	0.018
Average WSS, Pa	− 0.6 (− 2.1 to 0.5)	− 1.8 (− 3.5 to − 0.8)	0.006	− 1.29 (− 1.97 to − 0.61)	0.003
Peak WSS, Pa	− 5.7 (− 18.0 to 5.3)	− 11.5 (− 27.3 to 2.9)	0.16	− 6.67 (− 12.03 to − 1.29)	0.016
Systolic EL (AAo), mW	− 9.5 (− 20.5 to − 2.1)	− 6.7 (− 18.1 to − 0.22)	0.51	− 2.57 (− 8.24 to 3.10)	0.37
Diastolic EL(AAo), mW	− 0.7 (− 3.3 to 1.6)	− 0.1 (− 1.7 to 3.3)	0.28	0.35 (− 3.03 to 3.73)	0.84
Systolic EL (LV), mW	0.2 (− 1.4 to 3.2)	0.2 (− 0.4 to 2.5)	0.83	− 0.65 (− 2.88 to 1.58)	0.56
Diastolic EL (LV), mW	0.6 (− 2.7 to 3.5)	− 0.5 (− 2.0 to 1.9)	0.39	− 1.88 (− 4.72 to 0.97)	0.19

Continuous variables are presented as means ± standard deviations if normally distributed, and median (interquartile range) if not normally distributed. An analysis of covariance (ANCOVA) was performed using the individual pre-TAVR values of 4D flow CMR as covariates. a, P-value for the Mann–Whitney test; b, least squares mean difference using ANCOVA; c, P-value for ANCOVA. The other abbreviations are the same as those in Tables 1 and 3

### WSS in the AAo

**Fig. 4:**
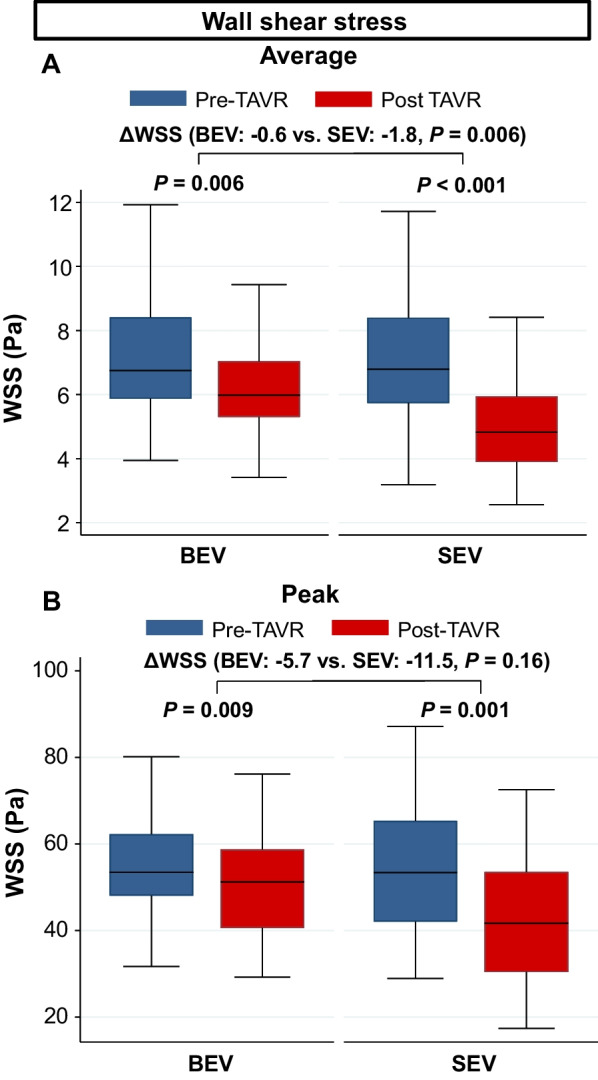
Average WSS (**A**) and peak WSS (**B**) in the entire AAo before and after TAVR in the BEV and SEV. *AAo* ascending aorta, *BEV* balloon-expandable valve, *SEV* self-expandable valve, *TAVR* transcatheter aortic valve replacement, *WSS* wall shear stress

**Fig. 5:**
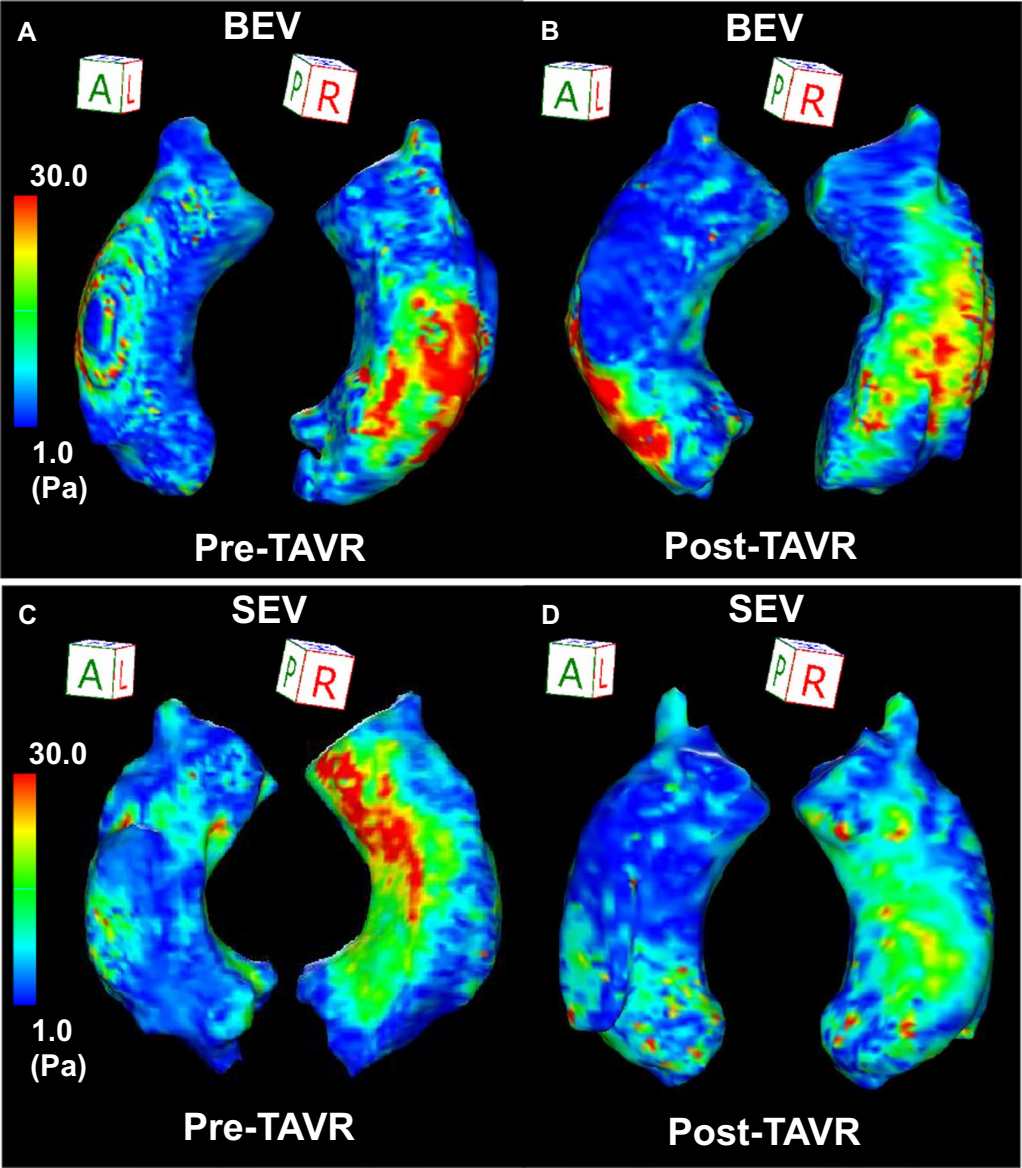
Representative cases of distribution of the peak WSS in the entire AAo before (**A**: BEV, **C**: SEV) and after (**B**: BEV, **D**: SEV) TAVR. *BEV* balloon-expandable valve, *SEV* self-expandable valve, *TAVR* transcatheter aortic valve replacement

**Fig. 6:**
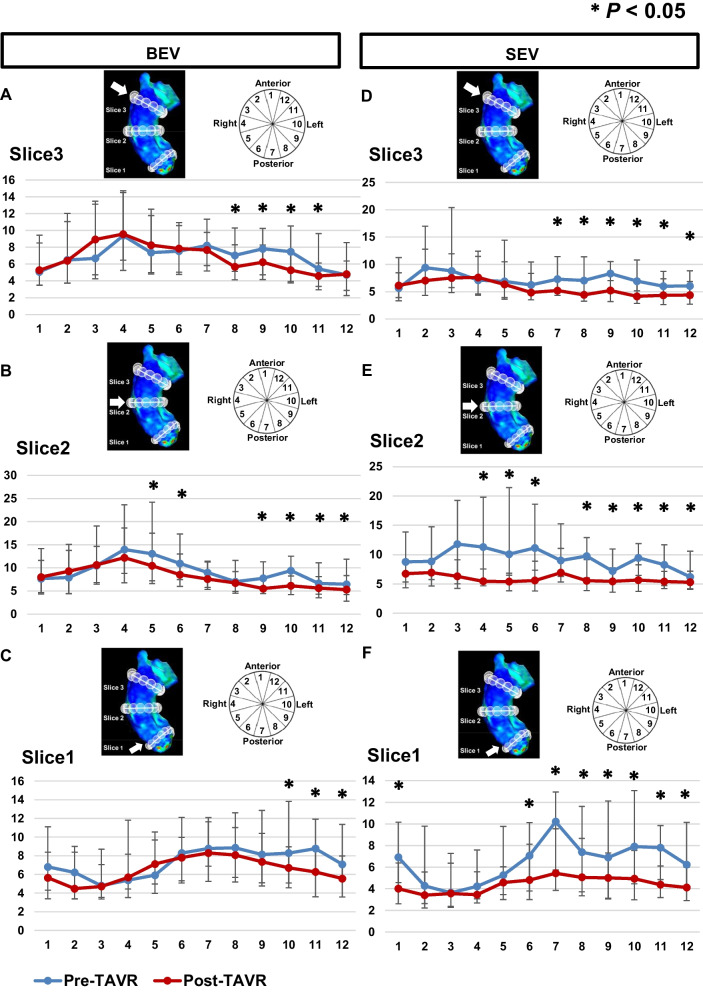
Distribution of the peak WSS in 12 segments along the aortic circumference for slice 3 (**A**: BEV, **D**: SEV), slice 2 (**B**: BEV, **E**: SEV), and slice 1 (**C**: BEV, **F**: SEV) before and after TAVR. *P < 0.05. *BEV* balloon-expandable valve, *SEV* self-expandable valve, *TAVR* transcatheter aortic valve replacement, *WSS* wall shear stress

There were no significant differences in the average and peak WSSs before TAVR in the BEV and SEV groups (Table 3). Changes in the average and peak WSSs in the entire AAo before and after TAVR are shown in Fig. 4. Average WSS significantly decreased after TAVR in the BEV and SEV groups (BEV: from 6.8 [5.9–8.4] Pa to 6.0 [5.3–7.0] Pa, *P* = 0.006, SEV: from 6.8 [5.7–8.4] Pa to 4.8 [3.9–5.9] Pa, *P* < 0.001). Conversely, the change observed in average WSS after TAVR was significantly higher in the SEV group compared to the BEV group (BEV: − 0.6 [− 2.1 to 0.5] Pa vs. SEV: − 1.8 [− 3.5 to − 0.8] Pa, *P* = 0.006). The peak WSS significantly decreased after TAVR in both groups, and there were no significant differences in the change observed in the peak WSS after TAVR between the groups (BEV: − 5.7 [− 18.0 to 5.3] Pa vs. SEV: − 11.5 [− 27.3 to 2.9] Pa, P = 0.16). In the ANCOVA, changes in the peak and average WSS were significantly higher in the SEV group compared to those in the BEV group (Table 5). Two representative cases that showed significant changes in the average WSS after TAVR are demonstrated in Fig. 5. Figure 6 shows peak WSS in 12 segments along the aortic circumference for analysis in plane slices 1 to 3 before and after TAVR. The WSS in the BEV group significantly decreased in the left and left anterior walls, while that in the SEV group was significantly decreased in the left, left anterior wall, and posterior wall.

### Energy loss in the ascending aorta and left ventricle

**Fig. 7:**
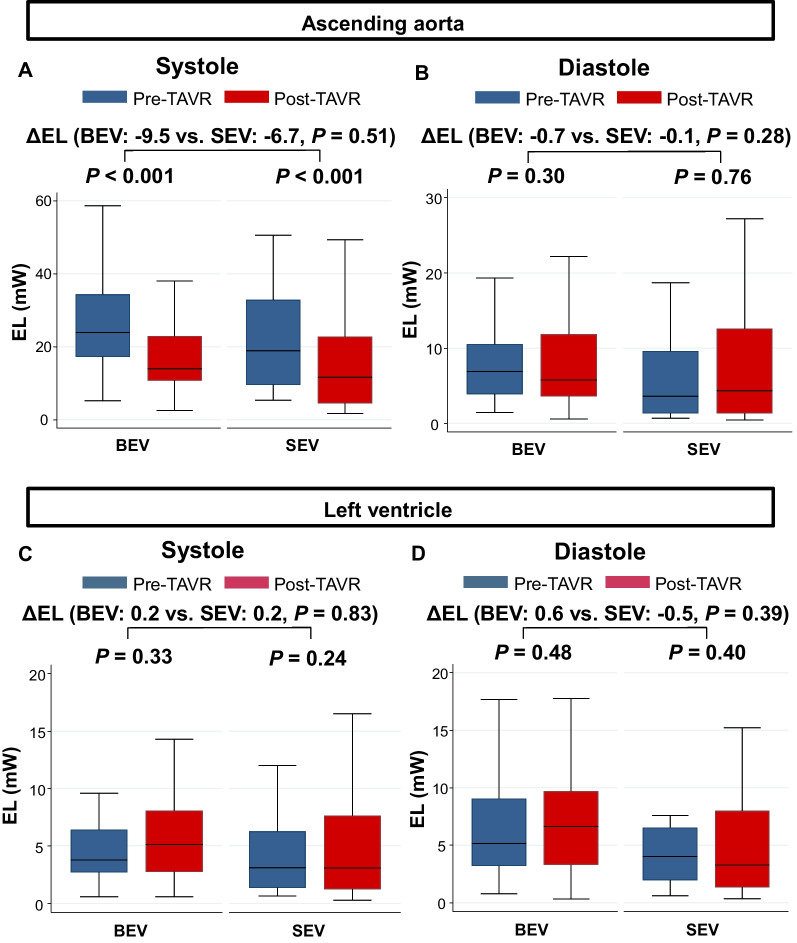
EL in the AAo before and after TAVR during (**A**) systole and (**B**) diastole; EL in the LV before and after TAVR during (**C**) systole and (**D**) diastole. *AAo* ascending aorta, *BEV* balloon-expandable valve, *EL* energy loss, *LV* left ventricle, *SEV* self-expandable valve, *TAVR* transcatheter aortic valve replacement

Changes in EL in the AAo and LV before and after TAVR are shown in Fig. 7. Systolic EL in the AAo significantly decreased after TAVR in both the BEV and SEV groups, whereas there were no significant differences in the number of changes in the systolic EL between groups. The diastolic EL in the AAo and systolic and diastolic EL in the LV were not significantly changed after TAVR in both the groups. These results were similar to those of the ANCOVA (Table 5).

## Discussion

This study is an initial report that evaluates pre- and early post-procedural differences in blood flow dynamics between BEV and SEV placement in patients who underwent TAVR. The major findings of this study were as follows. (1) Helical blood flow and flow eccentricity significantly decreased in the SEV group compared to the BEV group after TAVR; (2) Although the average WSS significantly decreased in both groups, its absolute reduction was significantly greater in the SEV group compared to the BEV group, and (3) Systolic EL significantly decreased after TAVR in both groups, while the absolute reduction in systolic EL was comparable between the two groups.

Previous studies revealed the role of helical blood flow in the AAo, indicating that the fairly coherent turning of blood may avoid excessive dissipation of energy by limiting flow instability in the arteries [20, 21]. Although helical blood flow plays a physiological role in facilitating blood transport, flow patterns change with the occurrence of morphological changes, such as in the case of AS [21]. Furthermore, helical flow grade is significantly associated with the severity of AS due to aortic valve calcific fusion and reduced mobility of blood. We previously reported that the degree of helical blood flow was higher in patients with AS compared to a normal patient group; however, it decreased significantly after TAVR [13]. In the present study, we found that the absolute reductions in helical blood flow and flow eccentricity grade in the AAo after TAVR were significantly higher in the SEV group compared to the BEV group. This may be attributed to the significantly larger valve in the SEV group compared to the BEV group in this study. Generally, in patients with the same aortic annular area, the size of the SEV is larger than that of the BEV. This is due to the supra-annular position of the valve attachment site of the SEV [5]. However, the results may also reflect differences in stent valve design. A previous computer simulation study on BEV for bicuspid AS demonstrated the tendency of BEV devices to expand asymmetrically in the aortic root [22]. Furthermore, another study showed differences between anatomical conformability of BEV versus SEV [23]. Notably, the BEV is characterized by high radial strength in the implanted host due to its high material strength. When deployed, the BEV undergoes local plastic deformation that keeps the device enlarged and in contact with the aortic wall, leading to a more elliptical shape of the device at the aortic bicuspid anatomy compared with the SEV device. In contrast, SEV are characterized by higher conformability because of the superelastic behavior of the nitinol stent material. Thus, stiff calcified plaque likely limits the opening of the SEV, which will then have a more circular shape when compared with the BEV device. Although we have excluded patients with bicuspid aortic valve in this study, the same phenomenon may be seen in severely calcified tricuspid aortic valves. Given this information, the different morphology of the THVs may contribute to differences in blood flow dynamics in the AAo.

In the present study, average WSS in the AAo after TAVR was significantly lower and the amount of change in average WSS was higher in the SEV group compared to the BEV group. Von Knobelsdorff-Brenkenhoff, et al. reported that abnormal blood flow patterns and flow eccentricity caused an increase in WSS by friction against the vessel wall and viscous dissipation [12]. Moreover, several studies using 4D flow CMR showed that abnormal WSS in the AAo, even in the absence of aortic valve stenosis or dilation, was primarily due to increased circumferential WSS [24–26]. In our study, WSS might might have been lower in the SEV group due to a significant decrease in the helical blood flow after TAVR compared to that in the BEV group. Notably, in the AAo, regional increases in WSS are associated with extracellular matrix dysregulation and elastic fiber thinning [27]. Guala A, et al. also reported that WSS, particularly its circumferential component, was an independent predictor of progressive dilation of the AAo in patients with a bicuspid aortic valve without significant valvular dysfunction [28]. These findings suggest that abnormal WSS after TAVR would lead to subsequent aortic degeneration during the long-term period.

Although parameters describing helical blood flow patterns and WSS can be used to quantitatively assess local blood flow structure, a global parameter that can be used to estimate an unfavorable blood flow structure is required when considering the pathophysiology of heart diseases. EL is considered as the loss of blood flow energy due to viscous friction in turbulent diseased flow, and it is assumed to be an important parameter to evaluate the cardiac workload [7]. EL is independent of existing heart failure or cardiac remodeling state such as the chamber size or ventricular wall motion; instead, it is expected to be a predictor of ventricular deterioration in the highly burdened state due to a cardiac disease [9, 29]. Our previous study demonstrated that patients with severe AS had higher systolic EL in the AAo than did the healthy participants. Furthermore, systolic EL in the AAo significantly decreased after TAVR [13]. The present study obtained similar findings, and furthermore, there were no significant differences in the changes in the systolic EL in the AAo between the BEV and SEV groups. These findings indicate that TAVR provides efficient blood flow dynamics and reduces the LV afterload regardless of the THV type.

### Clinical implications

An abnormal blood flow pattern and increased WSS in the AAo are indications of progression of an aortic disease [10]. Therefore, an improved blood flow pattern and reduced WSS after TAVR may reduce cardiovascular events in the long term. In particular, the indication for TAVR is expanded to relatively young patients with AS [30], and the evaluation of hemodynamics using 4D flow CMR would be useful. In addition, accurate assessment of blood flow dynamics in patients undergoing TAVR using 4D flow CMR would guide the selection of hemodynamically appropriate THV types. More specifically, the SEV may be the preferred THV in patients with AS and a strong degree of helical blood flow, flow eccentricity, and/or high WSS in the AAo before TAVR. Further studies are warranted to confirm whether impaired blood flow dynamics including the flow patterns, WSS, and EL assessed using 4D flow CMR are associated with adverse clinical events after TAVR, and whether these assessments are useful for selecting the type of THV in patients with AS undergoing TAVR.

In addition, the assessment of blood flow dynamics of patients with AS by using 4D flow CMR has the potential to address limitations of existing modalities such as echocardiography. In the present study, mean pressure gradient after TAVR was significantly higher in the BEV group compared to the SEV group. This result is consistent with those of previous studies [31, 32]. It is possible that some of these gradients after TAVR may be the artifacts of pressure recovery or assumptions inherent to the simplified Bernoulli equation, as opposed to true patient-prosthesis mismatch or valve dysfunction [31–33]. If there are more substantial gradients after TAVR, caution should be exercised while relying on echocardiographic Doppler assessment alone to diagnose true obstruction, given its potential for overestimation of the true gradient. Evaluation with 4D flow CMR can compensate for this limitation. In addition, it should be noted that the post-TAVR EOAI and SVI measured using echocardiography are susceptible to errors due to an increased blood flow in the LV outflow tract caused by the presence of the THV stent structure [34].

### Study limitations

There are limitations of this study that should be acknowledged. First, selection of THV type for TAVR was not randomized. As described in Methods, the appropriate valve type for patients undergoing TAVR was selected at a multidisciplinary Heart Team meeting after considering the examination findings.. Although it is unavoidable from a safety standpoint, the effect of selection bias cannot not be excluded. Second, the spatial and temporal resolutions of the 4D flow CMR were low in this study compared to those in the previous studies. In the setting of spatial and temporal resolutions, there is a trade-off between measurement duration and accuracy of parameters such as flow rate and WSS [35]. Reducing spatial or temporal resolution to shorten scan time adversely affects the accuracy of flow quantification and visualization, leading to underestimation of the WSS [36]. In this study, a low optimized in-plane spatial resolution (1.6 × 1.8 mm^2^) was used to improve the accuracy of WSS measurements. The slice gap was 2.0–2.5 mm to minimize the effect of anisotropic voxels. All acquisitions were made with the same imaging setting and analyzed with the same methodology both pre- and post-TAVR. Because this study focused on changes in parameters before and after TAVR placement, underestimation of WSS might be a less important. Third, the VENC was set with priority given to velocity to noise ratio in the AAo [37]. It is necessary to consider the effects of aliasing artifacts around the aortic valve. However, due to challenges in imaging and correcting aliasing caused by dephasing around the valve, we could not correct it. Consequently, inaccuracies in velocity, WSS, and EL around the valve are a limitation. In addition, the use of a high VENC setting for the systolic phase may fall within the range of noise in the diastolic phase. Fourth, the blood flow pattern evaluations may have differed among the observers, which is a limitation of the visual semi-quantitative method. Herein, the blood flow patterns were evaluated after thorough confirmation of the evaluation methods by two radiologists and one cardiologist. Although those assessments were reproducible in this study as our previous study [13], the influence of observer bias cannot be completely ruled out. In addition, evaluators were not completely blinded to patient information which may have resulted in observer bias. Fifth, we used echocardiography rather than CMR to assess LV remodeling, such as LVDD, LVMI, and LVEF, in this study. CMR is preferable for research and specific clinical conditions requiring higher accuracy and reproducibility than that offered by echocardiography. However, echocardiographic parameters were primarily used in this study to reduce examination time because the CMR parameters could not be evaluated adequately.

## Conclusions

Helical flow and average WSS in the AAo were significantly lower after TAVR in patients who received a SEV compared to those in whom a BEV was used. The SEV may be the preferred choice in patients with AS and a strong degree of helical blood flow and/or high WSS in the AAo before TAVR. Our findings provide functional insights into blood flow dynamics for valve selection in patients with AS undergoing TAVR. Further large-scale studies are needed to confirm the impact of hemodynamic differences on long-term prognosis in these patients.

## Data Availability

The datasets used during the current study are available from the corresponding author on reasonable request.

## Abbreviations

3D: Three-dimensional
4D: Four-dimensional
AAo: Ascending aorta
AS: Aortic stenosis
ANCOVA: Analysis of covariance
AVA: Aortic valve area
AVAI: Aortic valve area index
BEV: Balloon-expandable valve
CMR: Cardiovascular magnetic resonance
CT: Computed tomography
E/A: The ratio between early and late diastolic transmitral flow velocity
EL: Energy loss
EOA: Effective orifice area
EOAI: Effective orifice area index
HF: Heart failure
IQR: Interquartile range
LA: Left atrial
LAVI: Left atrial volume index
LV: Left ventricular
LVDD: Left ventricular end-diastolic dimension
LVEF: Left ventricular ejection fraction
LVMI: Left ventricular mass index
mPG: Mean transaortic pressure gradient
MRI: Magnetic resonance imaging
SEV: Self-expandable valve
TAVR: Transcatheter aortic valve replacement
THV: Transcatheter heart valve
VENC: Velocity encoding
WSS: Wall shear stress
